# Correlation between gene expression and MRI STIR signals in patients with chronic low back pain and Modic changes indicates immune involvement

**DOI:** 10.1038/s41598-021-04189-5

**Published:** 2022-01-07

**Authors:** Maria Dehli Vigeland, Siri Tennebø Flåm, Magnus Dehli Vigeland, Ansgar Espeland, Per Martin Kristoffersen, Nils Vetti, Monica Wigemyr, Lars Christian Haugli Bråten, Elisabeth Gjefsen, Elina Iordanova Schistad, Anne Julsrud Haugen, Anne Froholdt, Jan Sture Skouen, John-Anker Zwart, Kjersti Storheim, Linda Margareth Pedersen, Benedicte Alexandra Lie, the AIM Study Group, Audny Anke, Bendik Slagsvold Winsvold, Britt Elin Lurud, Christian Hellum, Erling Andersen, Fredrik Granvigen, Gunn Hege Marchand, Guro Kjos, Hege Andersen, Hilde Presberg, Ida Beate Østhus, Jens Ivar Brox, Jörg Aßmus, Karianne Wiger Gammelsrud, Knut Morten Huneide, Lars Grøvle, Mads Peder Rolfsen, Maja Wilhelmsen, Margreth Grotle, Marianne Thorsø, Olav Lutro, Øystein Petter Nygaard, Sigrun Randen, Siv Krüger Claussen, Terese Fors, Thomas Istvan Kadar, Thor Einar Holmgard, Veronica Sørensen, Vidar Rao

**Affiliations:** 1 Division of Clinical Neuroscience, Department of Research, Innovation and Education, Oslo University Hospital, Oslo, Norway; 2 Present Address: Faculty of Medicine, University of Oslo, Oslo, Norway; 3 Department of Medical Genetics, Oslo University Hospital, Oslo, Norway; 4 Department of Radiology, Haukeland University Hospital, Bergen, Norway; 5 Department of Clinical Medicine, University of Bergen, Bergen, Norway; 6 Department of Physiotherapy, Oslo Metropolitan University, Oslo, Norway; 7 Department of Physical Medicine and Rehabilitation, Oslo University Hospital, Oslo, Norway; 8 Department of Rheumatology, Østfold Hospital Trust, Grålum, Norway; 9 Department of Physical Medicine and Rehabilitation, Drammen Hospital, Drammen, Norway; 10 Department of Physical Medicine and Rehabilitation, Haukeland University Hospital, Bergen, Norway; 11 Department of Global Public Health and Primary Care, University of Bergen, Bergen, Norway; 12 Department of Rehabilitation, University Hospital of North Norway, Tromsø, Norway; 13 Department of Clinical Medicine, Faculty of Health Sciences, UiT The Arctic University of Norway, Tromsø, Norway; 14 Department of Physical Medicine and Rehabilitation, St. Olavs Hospital, Trondheim University Hospital, Trondheim, Norway; 15 Department of Orthopaedic Surgery, Oslo University Hospital, Oslo, Norway; 16 Department of Clinical Engineering, Haukeland University Hospital, Bergen, Norway; 17 Faculty of Medicine and Health Sciences, Norwegian University of Science and Technology, Trondheim, Norway; 18 National Advisory Unit of Spinal Surgery, Trondheim, Norway; 19 Department of Neurology, Vestre Viken Hospital, Drammen, Norway; 20 Centre for Clinical Research, Haukeland University Hospital, Bergen, Norway; 21 Department of Microbiology, Oslo University Hospital, Oslo, Norway; 22 Department of Physical Medicine and Rehabilitation, Østfold Hospital Trust, Grålum, Norway; 23 Stavanger University Hospital, Stavanger, Norway; 24 Department of Neurosurgery, St. Olavs Hospital, Trondheim University Hospital, Trondheim, Norway; 25 Norwegian Back Pain Association, Drammen, Norway

**Keywords:** Gene expression, Disease genetics, Chronic pain, Musculoskeletal system, Osteoimmunology

## Abstract

Disability and distress caused by chronic low back pain (LBP) lacking clear pathoanatomical explanations cause huge problems both for patients and society. A subgroup of patients has Modic changes (MC), identifiable by MRI as vertebral bone marrow lesions. The cause of such changes and their relationship to pain are not yet understood. We explored the pathobiology of these lesions using profiling of gene expression in blood, coupled with an edema-sensitive MRI technique known as short tau inversion recovery (STIR) imaging. STIR images and total RNA from blood were collected from 96 patients with chronic LBP and MC type I, the most inflammatory MC state. We found the expression of 37 genes significantly associated with STIR signal volume, ten genes with edema abundancy (a constructed combination of STIR signal volume, height, and intensity), and one gene with expression levels significantly associated with maximum STIR signal intensity. Gene sets related to interferon signaling, mitochondrial metabolism and defense response to virus were identified as significantly enriched among the upregulated genes in all three analyses. Our results point to inflammation and immunological defense as important players in MC biology in patients with chronic LBP.

## Introduction

Activity limitation and work absence caused by low back pain (LBP) has emerged as an enormous economic, societal, and social burden globally^1,2^. A large proportion of LBP patients (10–70%) will develop chronic LBP, defined as duration of pain for longer than 3 months^3,4^. Despite considerable research efforts, LBP remains poorly understood and has in most cases unknown pathoanatomical cause^5^.

Increasing attention, both in research and clinical settings, has been directed towards patients with Modic changes (MC) as a subgroup among LBP patients^6^. MC are vertebral bone marrow lesions identifiable by magnetic resonance imaging (MRI) first described in the late 80’s^7,8^. An association between MC and LBP has been suggested^9,10^, but conclusions after several studies are inconsistent^11^. Three types of MC are distinguished by their intensity on T1- and T2-weighted MRI. MC type I is considered to represent the most inflammatory state^12,13^ with possible association to LBP^14^. The extent of edema in MC of type I can be evaluated using short tau inversion recovery (STIR). This MRI technique suppresses the signals from fat, allowing for a more sensitive detection of edema signals, and has been suggested as a supplement to the routinely used T1- and T2-weighted scans^15^.

The etiology behind MC is unknown. Prevailing theories emphasize an immunological component, involving autoimmunity or low-grade bacterial infections in the intervertebral disc, triggering a detrimental cascade of inflammation in the vertebrae^16^. To advance in the search for biological explanations and treatment options to subjective conditions like unspecific back pain, unbiased endpoints can be useful. Analysis of gene expression profiles from blood reflects systemic processes, potentially shedding light on the immunological state and underlying pathology of a disorder. In this study, we aimed at improving the pathobiological understanding of MC type I related STIR signal increases in patients with chronic LBP, using whole blood gene expression profiling and pathway analysis.

## Materials and methods

### Study cohort

The study is based on baseline data from a sub-sample of patients with chronic LBP and MC included in a randomized controlled trial comparing amoxicillin to placebo (the Antibiotics in Modic changes (AIM) study, n = 180)^17^. The patients had suffered from LBP with a duration of more than 6 months and had MC type I or II in the vertebral body at the same disc level as a lumbar disc herniation verified on MRI within the preceding 2 years. Patients were excluded from the study if a specific diagnosis could explain their low back symptoms (e.g., tumor or spinal stenosis), if they had undergone surgery for disc herniation the previous 12 months or had former low back surgery for reasons other than disc herniation. Eligibility criteria and the study protocol for the AIM study is fully published elsewhere^18^.

Out of 118 patients with MC type 1, we included in this study patients of Caucasian ethnicity with successful blood sampling at screening (n = 96).

Blood samples and STIR images were collected for all patients before randomization and start of intervention in the AIM study. Data regarding age, gender, education, body mass index (BMI), ethnicity, smoking habits, work and physical activity habits were gathered by trial care givers (medical doctors, physiotherapists or study nurses). Additionally, information was collected on back pain history, current disability (Roland-Morris Disability Questionnaire (RMDQ), 0–24, where 0 corresponds to no disability and 24 corresponds to maximum disability^19^), emotional distress (Hopkins Symptom Checklist-25, where values ≥ 1.75 associate with psychiatric diagnosis^20^) and fear-avoidance beliefs (Fear-Avoidance Beliefs Questionnaire (FABQ), where higher values indicate more fear avoidance beliefs^21^).

Written informed consent was obtained from all patients in accordance with the Helsinki Declaration, and the study was approved by the Regional Ethics Committee in South East Norway (project 2014/158).

### MRI

MRI was performed at the local study sites using the same MRI protocol and scanner type (Avanto 1.5 T, Siemens). The imaging included standard T1- and T2 weighted fast spin echo images and sagittal STIR images, as detailed previously^22^.

MC were defined as type I (hypointense T1 signal and hyperintense T2 signal), type II (hyperintense T1 signal and iso/hyperintense T2 signal) and type III (hypointense T1 and T2 signal). MC with diameter ≤ 5 mm or height < 10% of vertebral body height did not qualify the patient for inclusion. Patients with MC type I at the endplate superior and/or inferior to the previously herniated disc level were included in the present study also if they had other MC types at the same or a different endplate. Disc herniation was defined as a focal displacement of disc material, i.e. involved < 25% of the disc circumference^23^. In this study, disc herniation was further classified as protrusion (if its largest diameter was smaller than its base) or extrusion (base smaller than diameter—or sequestration)^23^. Disc degeneration (DD) was graded using Pfirrmann grades, which range from 1 (no DD) to 5 (highest degree of DD)^24^.

MC related STIR signal increase was assessed at 12 endplates (Th12-S1)^22^. Three endpoint variables were defined prior to analysis: (I) Total volume of STIR signal increase (henceforth referred to as “STIR volume”). Each endplate was given a score based on the volume of increased STIR signal in percent out of the total vertebral body volume (0: no STIR signal, 1: < 10%, 2: < 25%, 3: 25–50%, 4: > 50%). The total sum score for the 12 endplates was reported (possible values 0–48). (II) Maximum STIR signal intensity (henceforth “STIR intensity”). The maximum intensity of MC related STIR signal increase at any endplate, reported as a percentage on an intensity scale ranging from normal vertebral body intensity (0%) to cerebrospinal fluid intensity (100%) (possible values 0–100%). (III) «STIR composite» was pre-constructed to categorize the patients according to the abundancy of edema at the level of the previously herniated disc^25^. STIR composite 1 represented MC related STIR signal increase with largest volume of < 25% and maximum intensity < 25%. STIR composite 3 represented STIR signal increase with volume ≥ 25% and height > 50% of vertebral body and maximum intensity ≥ 25% and presence at both sides of the disc. Patients in neither group were categorized as STIR composite 2.

Two radiologists independently evaluated the MRI images. Their inter-rater agreement was mostly very good for presence of MC type I (kappa 0.78–0.85 depending on level) and presence of STIR signal increase (0.83–0.90), and moderate for volume of STIR signal increase (0.47–0.61)^22^ and DD grades 3–4 vs 5 (0.44; grades 1–2 not reported). In the case of disagreements, they negotiated a conclusive finding (for presence of MC type I and DD grade) or a third radiologist was consulted, and the majority rating was used (for STIR findings). For the intensity measurements, the mean of two radiologists’ values was used. To further describe the samples, one experienced radiologist classified disc herniations as protrusions or extrusions.

The correlation between the STIR variables was examined using Pearson’s correlation coefficient (*r)*.

### Blood sample collection and treatment

Peripheral blood was sampled from each patient in Tempus Blood RNA Tubes (Thermo Fisher Scientific, Waltham, MA, USA), on average 14 days before the MRI (max 48 days). Simultaneously, blood samples were collected in EDTA tubes for 5-part differential blood cell count using flow cytometry locally at each study center, giving the relative content of neutrophils, lymphocytes, basophils, eosinophils, and monocytes in the samples. The relative blood cell counts were tested for correlation with the STIR variables.

### RNA isolation, preparation and sequencing

Total RNA was isolated from the Tempus Blood RNA Tubes using the Preserved Blood RNA Purification Kit I (Norgen Biotek, Thorold, Canada) according to the manufacturer’s instruction. DNAse treatment was carried out as recommended. The quality and concentration of the RNA were measured using the BioAnalyzer 6000 Nano kit (Agilent Technologies, Santa Clara, CA, USA) and Qubit RNA HS (Thermo Fisher Scientific, Waltham, MA, USA).

Ribosomal RNA (rRNA) and globin transcripts dominate the RNA pool of whole blood samples. rRNA provides negligible relevant information about the transcriptome, and the globin transcripts were not expected to be relevant in this study. The total RNA samples were therefore depleted for rRNA and globin transcripts to maximize the number of informative reads retrieved from high-throughput sequencing. The first 82 of the 96 RNA samples were depleted for rRNA and globin transcripts using the Globin-Zero Gold rRNA Removal Kit (Illumina, San Diego, CA, USA), before cDNA library preparation using the strand-specific TruSeq RNA-seq library prep (Illumina, San Diego, CA, USA). The depletion kit was discontinued and replaced by a kit combining the ribosomal- and globin depletion with the cDNA library preparation. The last 14 samples were therefore depleted and prepared using the new TruSeq Stranded Total RNA with Ribo-Zero Globin kit (Illumina, San Diego, CA, USA).

The depleted RNA samples were sequenced with 2 × 75 bp paired-end configuration on the HiSeq3000 platform (Illumina). The quality of the sequencing was assessed using FastQC and Qualimap^26,27^.

### Bioinformatics and analysis of differential gene expression

The raw sequencing data was trimmed for low quality reads and adapter sequences using Trim Galore! v0.4.5^28^. The reads were mapped to the human genome (GRCh38.p10) using HISAT2 v2.1.0^29^ and counted with featureCounts from Subread v1.6.3^30^, using gene coordinates from Ensembl 88^31^. Only autosomal genes with a read count of more than three in at least five subjects were kept for further analyses.

Analysis of differential expression was performed with the R package DESeq2 v1.32.0^32^. The DESeq2 pipeline includes normalization of the samples by sequencing depth and RNA composition, using the implemented median of ratios method. The differential expression analysis is done by fitting a negative binomial model to each gene and applying a Wald test to the log2 fold change between groups.

We tested for associations between gene expression and the three STIR variables separately (STIR volume, STIR intensity and STIR composite), adjusting for sequencing batch effects, age and gender. The analyses were repeated, adjusting for white blood cell proportions. The p-value distributions were estimated empirically from the z-scores reported by DESeq2, using the R package fdrtool v1.2.15^33^, controlling the false discovery rate (FDR) with the Benjamini–Hochberg method^34^.

### Gene set enrichment analysis

To explore whether the patients’ gene expression profiles reflect the deregulation of certain biological pathways, we performed gene set enrichment analysis on the ranked gene lists from the differential expression analysis. We used publicly available enrichment map gene sets from the Bader lab (version from March 01, 2020) containing both manually curated and computationally generated gene sets^35^. 23 custom autoimmunity gene sets were created from Genome-wide association study (GWAS) data (Table S1), and a total of 18,577 gene sets were used as input to the GSEA analyses. 5474 gene sets were left for the analyses after gene set size filtering.

The GSEA v4.1.0 software from UC San Diego and Broad Institute^36,37^ was used for the gene set enrichment analyses. GSEA searches for pathways whose genes are significantly enriched at the top or bottom of a given ranked gene list. The input gene lists contained all genes from the differential expression analyses, ranked according to significance and odds ratio: The most significantly upregulated genes found on top of the list, and the most significantly downregulated genes on the bottom. Following general recommendations, gene sets containing less than 15 or more than 200 genes were filtered out from the analyses^38^. The GSEA results were visualized using Cytoscape v3.8.2^39^ with apps EnrichmentMap v3.2.1^35^ and AutoAnnotate v1.3.3^40^. The enrichment map was created with parameters FDR *q* < 0.05, and Jaccard Overlap combined coefficient > 0.375 with combined constant = 0.5.

### Interferon-regulated genes

We investigated whether the significantly differentially expressed genes from our analyses were recognized as interferon-regulated genes using the Interferome database v2.01^41^. The database contains data from microarray experiments of murine or human cells treated with interferon in vivo or in vitro, summarizing observed effects of interferon stimulation on the expression of genes. The search was restricted to experiments on normal blood (i.e., without a known genetic condition, disease state or pre-treatment), using the differentially expressed genes significant at FDR < 0.05 from all analyses.

## Results

### Patient characteristics and STIR variables

**Figure 1:**
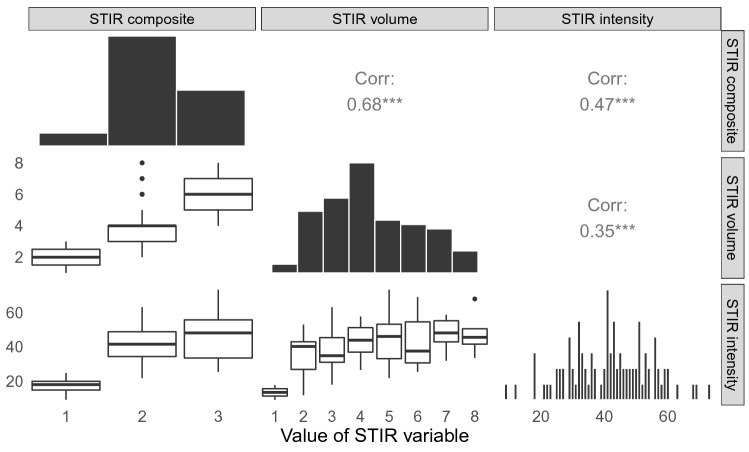
STIR variable distributions and Pearson correlations. Corr: Pearson’s r. ***p < 0.001.

The present study included 96 patients with LBP and MC type I (age between 27 and 63 years, 61 women). The patients had an average LBP intensity of 6.5 on a 0–10 numerical rating scale (NRS) and LBP lasting for a median of 3 years (Table 1). Most patients (92 of 96) had DD grade 4 or 5. The distribution of the STIR volume, STIR intensity and STIR composite variables in the study sample is illustrated in Fig. 1. The measured ranges of STIR volume and intensity were 1–8 and 9–73, respectively. There was weak to moderate correlation between the variables, with the strongest correlation found between STIR volume and STIR composite (0.68, p = 1.6 × 10^–14^).

**Table 1:** Characteristics of the 96 patients included in the study.

Variable	Value
Female (%)	61 (63.5)
Age (mean (SD))	45.3 (8.7)
BMI (median (IQR)), n = 93	24.5 (4.8)
Smoking (%), n = 91	23 (25.3)
LBP intensity, NRS (mean (SD)), n = 92	6.5 (1.1)
LBP duration in years (median (IQR)), n = 92	3.0 (5.0)
Disability, RMDQ (mean (SD)), n = 88	12.5 (4.0)
Fear avoidance beliefs, FABQ—physical activity (mean (SD)), n = 92	11.9 (5.8)
Fear avoidance beliefs, FABQ—work (mean (SD)), n = 91	15.8 (11.0)
Emotional distress, HSCL-25 > 1.75 (%), n = 92	19 (20.7)
**Educational level (%), n = 92**	
Primary school (9 years)	8 (8.7)
High school (12 years)	10 (10.9)
College or university (< 4 years)	50 (54.3)
University (≥ 4 years)	24 (26.1)
**Physical workload (%), n = 93**	
Mostly sitting	38 (40.9)
Job requires a lot of walking	21 (22.6)
Job requires a lot of walking and lifting	15 (16.1)
Job requires physically heavy work	4 (4.3)
Not working^†^	15 (16.1)
**Disc degeneration, highest Pfirrmann grade (%)**	
Grade 3	4 (4.2)
Grade 4	50 (52.1)
Grade 5	42 (43.8)
**Disc herniation past two years, type (%)**	
Protrusion(s) only	52 (54.2)
Extrusion	44 (45.8)

*SD* Standard deviation, *BMI* Body mass index, *IQR* Interquartile range, Q3-Q1, *LBP* Low back pain, *NRS* Numerical rating scale, *RMDQ* Roland-Morris Disability Questionnaire, *FABQ* Fear-Avoidance Beliefs Questionnaire, *HSCL-25* Hopkins Symptom Checklist-25. ^†^On sick leave, disabled, unemployed or studying.

### Cell type proportions

All patients had either normal range or minutely increased or decreased blood cell counts, with no suspicion of underlying infection or pathology/comorbidities (Figure S1). None of the cell type proportions correlated significantly with any of the three STIR variables (Figure S2).

### Quality control of gene expression data

The isolated total RNA had a mean RNA integrity number (RIN) of 9.2 and concentration of 157 ng/μl. After RNA sequencing, 96.6% of the reads were aligned to the reference genome, and 53.4% of these were assigned to genes. The unassigned proportion was mostly multimapped or ambiguously mapped reads.

Lowly expressed genes were filtered out, removing less credible counts and reducing the number of tests in the analyses. Reads from 25,944 genes (58% coding for proteins) represented by more than three counts in at least 5 samples were retained. The two different globin depletion kits used during the sequencing library preparation caused a small batch effect in the gene expression profiles (Figure S3). This was accounted for through the flow cell batch effect adjustment in the differential expression analyses.

### Differential expression analyses

**Figure 2:**
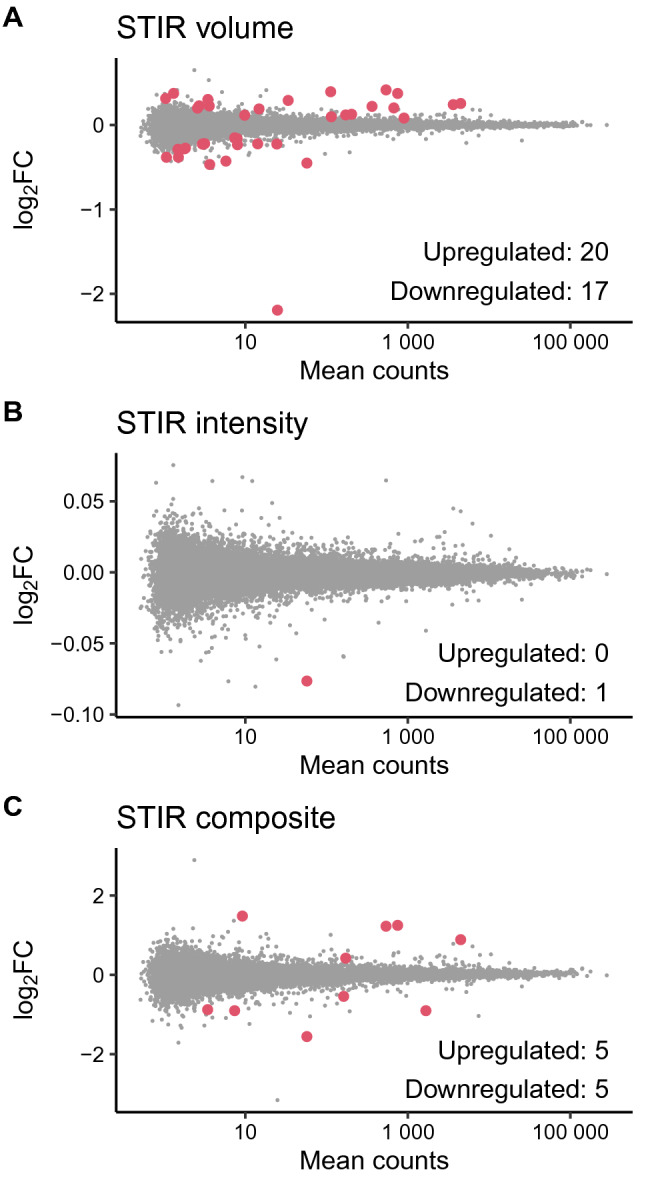
MA plots showing the differential expression of all investigated genes associated with (**A**) STIR volume, (**B**) STIR intensity, (**C**) STIR composite. Each point represents a gene, red points correspond to significantly differentially expressed genes (padj < 0.05). Log2FC: Log2 fold change of gene expression. Mean counts: Average of read counts for a gene across all samples (normalized by sequencing depth).

In total 42 genes were found to be significantly up- or downregulated in association with the STIR variables (Fig. 2). The majority (45%) were protein coding genes. The rest were mainly long non-coding RNA (29%), other non-coding RNAs (14%) and pseudogenes (10%) (Figure S4).

We found 37 genes with expression levels significantly associated with STIR volume. One gene was associated with STIR intensity, while ten genes had differential expression associated with the STIR composite variable (FDR < 0.05, Table S2–4). The corresponding numbers of significantly associated genes using FDR < 0.10 were respectively 87, 3 and 21.

The most significantly differentially expressed protein coding genes resulting from the analysis of STIR volume, were *GLYATL2* (adjusted p-value (p_adj_) = 6.3e−04), *IFI27* (p_adj_ = 9.9e−04), *MYO18B* (p_adj_ = 5.6e−03) and *APOLD1* (p_adj_ = 7.7e−03). *GLYATL2* correlated negatively, while *IFI27*, *MYO18B* and *APOLD1* correlated positively with STIR volume. In the analysis of the composite STIR variable, *GLYATL2* (p_adj_ = 2.4e−08), *NPTX1* (p_adj_ = 6.3e−07) and *MYL9* (p_adj_ = 3.8e−06) were the most significantly differentially expressed genes, all three correlating negatively with increasing STIR composite value. The only gene significantly differentially expressed in the analysis of STIR intensity was *GLYATL2* (p_adj_ = 0.028).

**Figure 3:**
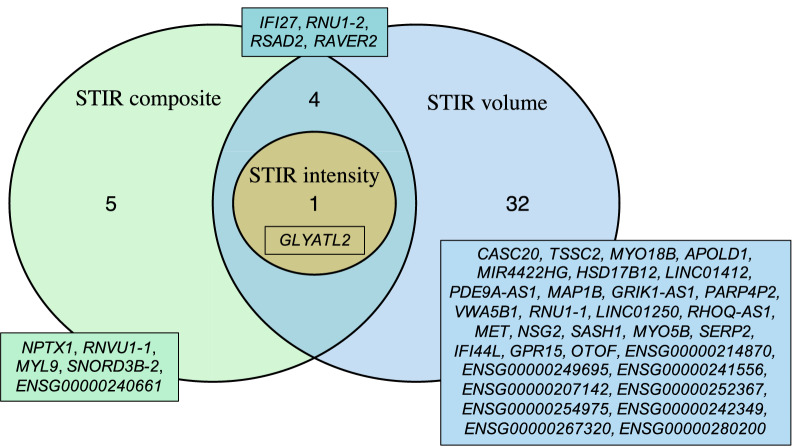
Venn diagram of overlapping DE genes from STIR analyses significant at FDR < 0.05.

There were overlapping results across the analyses (Fig. 3). *GLYATL2* was significant in all three analyses, while *IFI27*, *RNU1-2*, *RSAD2* and *RAVER2* were significantly differentially expressed in both the analysis of STIR volume and STIR composite (Figure S5).

### Gene set enrichment analyses

**Figure 4:**
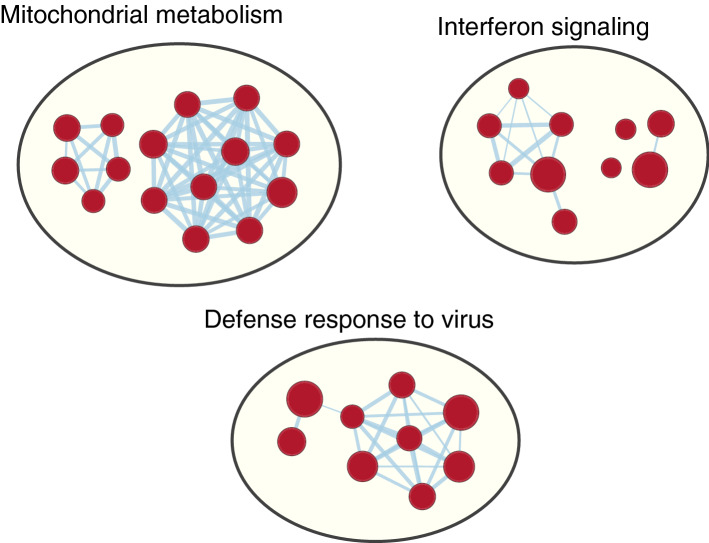
Gene sets significantly enriched among differentially expressed genes across all analyses, visualized using Cytoscape. Pathways related to mitochondrial metabolism, interferon signaling, and defense response to virus were significantly enriched among the upregulated genes in all three analyses. Each pathway is represented by a red node, overlaps of genes between the pathways are shown as light blue edges. The node size represents the number of genes in the gene set.

We next investigated whether certain biological pathways were enriched (FDR < 0.05) in the differentially expressed genes. Genes from 59 gene sets were found significantly enriched among the STIR volume associated upregulated genes (Table S5). The most strongly enriched pathways were related to interferon signaling and response (Fig. 4). Additionally, pathways involved in mitochondrial metabolism and responses to virus were among the enriched gene sets.

In the analysis of maximum STIR signal intensity, 775 gene sets were found significantly enriched among the up- and downregulated genes (Table S6). 235 of these were enriched among the upregulated genes. The strongest enrichment was again observed for interferon response and mitochondrial metabolism, as seen in the analysis of STIR volume, in addition to cell cycle related gene sets. 540 gene sets were enriched among the downregulated genes. Interleukin and cytoskeleton related pathways were among the most strongly enriched gene sets.

In the gene set enrichment analysis of STIR composite, 144 gene sets were enriched among the upregulated genes, none among the downregulated genes (Table S7). Immune-related gene sets predominated the list, together with mitochondrial metabolism (as observed in the previous two analyses), cell cycle and antigen presentation.

Exploring the overlap between the different STIR variables, 36 gene sets were found significantly enriched in all three gene set enrichment analyses (Fig. 4). More details can be found in Table S8.

### Cell type proportion adjustment

To investigate whether differences in the patients’ white blood cell proportions affect our results, we repeated all analyses adjusting for the flow cytometry-measured white blood cell proportions. The analysis results from the two approaches were mostly overlapping, albeit with fewer significant findings when adjusting for cell type proportions (Figure S5).

### Interferon-regulated genes

As interferon signaling was the pathway most significantly enriched among the differentially expressed genes, we investigated whether the significantly differentially expressed genes from our analyses were recognized as interferon-regulated genes in the Interferome database.

Nine of the in total 42 significantly differentially expressed genes (FDR < 0.05) were reported as observed up- or downregulated by interferon-α, β or γ with a fold change of at least 2 (Table 2).

**Table 2:** Interferon-regulated genes among the significantly differentially expressed genes.

Gene	IFN-α	IFN-β	IFN-γ	Our data
*HSD17B12*			−	+
*IFI27*	+	+	+	+
*IFI44L*	+	+	+	+
*MAP1B*	+			−
*MET*			+ /−	−
*MYL9*			−	−
*OTOF*		+		+
*RSAD2*	+	+	+	+
*SASH1*	+		+	+

The three first columns show whether the genes are reported as up ( +) or down (−) regulated by IFN-α, IFN-β or IFN-γ in the Interferome database v2.01. The last column correspondingly shows the direction of expression change observed in our data.

*IFN* Interferon.

## Discussion

To our knowledge, this is the first study exploring how MC-related STIR findings correlate with gene expression levels in patients with chronic LBP and MC type 1. Overall, the results implicated gene sets relevant to inflammation and immunological defense.

We identified multiple genes with expression levels significantly associated with the STIR signals. Several of the significant genes encode proteins involved in processes of potential importance to the development of MC.

Across all analyses, *GLYATL2* was significantly lower expressed with increasing STIR signals. This gene codes for Glycine N-acyltransferase-like 2, a protein involved in the synthetizing of N-acyl glycines^42^. Among the wide range of functions of these signaling molecules, they are believed to contribute to the resolution of inflammation and have pain-reducing effects^43,44^. A lower expression of this gene might therefore be connected to the chronicity of inflammation observed in MC patients. It is however to be noted that the association observed in our data could be driven by a very high expression of this gene in a few patients.

Three protein coding genes were additionally significantly differentially expressed both in the STIR volume and STIR composite analyses (*RAVER2, IFI27* and *RSAD2*). This overlap could partly be explained by the moderate correlation observed between the two STIR variables (Pearson's *r* = 0.68), but might also indicate that these are more robust findings. *RAVER2*, coding for Ribonucleoprotein, PTB Binding 2, is involved in alternative splicing regulation^45^. SNPs in this gene have been associated with Ulcerative colitis, an inflammatory bowel disease that causes long-lasting inflammation and ulcers in the digestive tract, suggesting a role for this gene in autoimmunity^46^. *IFI27*, coding for Interferon Alpha Inducible Protein 27, and *RSAD2*, coding for Radical S-Adenosyl Methionine Domain Containing 2, are both reported to be interferon-regulated genes. *IFI27* is implicated in cell apoptosis induced by DNA damage^47^, while *RSAD2* is demonstrated to be involved in a wide range of innate immune and antiviral responses^48^.

Among the non-overlapping results, the most significant finding in the differential expression analysis of STIR volume was the negative correlation of *CASC20*. This is a long non-coding RNA which function is still unknown, but variants in this gene have through GWAS been associated with height and body shape^49,50^. This is interesting, as related characteristics like BMI has been associated to MC^51^. Other genes with expression levels associated to STIR volume were *MYO18B*, which have been reported as relevant for muscle homeostasis and movement^52^, and *APOLD1*, coding for Apolipoprotein L domain containing 1, a protein thought to regulate endothelial cell signaling and vascular function in association with angiogenesis^53^. This finding agrees with the concept of vascular development in the subchondral bone marrow being a hallmark of MC type 1^54^.

In addition to *GLYATL2*, the gene with expression most significantly associated with the STIR composite variable was *NPTX1*, coding for Neuronal Pentraxin 1, a secreted protein involved in neural development and remodeling^55^. Neuronal Pentraxin 1 has been shown to regulate mitochondria-driven neuronal injury in mice, while knocking down the gene provide neuroprotection^56^. This might be noteworthy, as an increased nerve-ingrowth has been demonstrated in vertebral endplates with MC compared to normal endplates^57^.

There were no other significantly differentially expressed genes aside from *GLYATL2* associated to the STIR intensity variable. This low number of significant associations could be explained by the nature of the variable. This variable is a point value representing maximum observed STIR intensity and does therefore not reflect overall spread or amount of edema.

It should be emphasized that the genes implicated through expression levels associated with STIR variables in the present study need, in addition to replication, to be functionally studied, also at the protein level, in order to establish a biological role in MC type 1.

In addition to the previously mentioned interferon-regulated genes, the GSEA analyses revealed genes relevant to pathways of both interferon alpha (IFN-α) and interferon gamma (IFN-γ) responses to be significantly enriched among the differentially expressed genes. The interferons are signaling molecules with diverse roles in immunological defense and inflammation. Interferons have shown to mediate the activation and development of a range of immune cells and are involved in several autoimmune diseases^58^. Additionally, interferons have emerged as important players in bone metabolism, as studies from the field of osteoimmunology demonstrate how these molecules interfere with osteoclast differentiation^59^. Our study of MC affected vertebrae join a range of studies emphasizing the role of interferon dysregulation in spine conditions. In disc samples from patients with degenerated and herniated discs, a significant correlation was found between Pfirrmann DD grade (ranging from 2 to 5) and the gene expression of IFN-α1^60^. Through gene expression profiling of degenerative versus non-degenerative disc cells, interferon signaling has been shown to be the primarily dysregulated pathway. Upregulation of IFN-α-induced genes is hypothesized to accelerate disc degeneration through a negative regulation of the cell cycle leading to decreasing disc cell numbers^61,62^. When mechanically inducing degeneration of bovine intervertebral discs, signaling molecules including IFN-γ were found upregulated. These molecules were thought to drive activation of microglia in the spinal cord, increasing the upregulation of other neuroinflammatory markers, driving further disc degeneration^63^.

One of the principal theories concerning the development of MC involve autoimmunity. Disc material is considered immune privileged, and it is shown that nucleus pulposus cells in contact with immune cells from the bone marrow can trigger inflammatory autoimmune processes and MC^64^. Interferons and interferon-regulatory factors have been associated to a range of autoimmune conditions^58^, and interestingly, IFN-γ has been found highly expressed in nucleus pulposus tissue after disc herniation^65^, and in higher concentration in herniated discs with extruded nucleus pulposus tissue than in intact herniated discs^66^. MRI has moderate accuracy compared to surgery for whether disc herniations extrude through the annulus fibrosus and the posterior longitudinal ligament^67^. Still, it should be noted that many disc herniations in our sample were shaped as extrusions on MRI (Table 1).

Interferons might also be of importance for the development of chronic pain, which is suggested both for sciatica^68^ and neuropathic pain^69^. Levels of in vitro production of IFN-γ from blood have been positively correlated with pain scores in patients with nonspecific acute, but not chronic LBP^58^. Similarly in another study, IFN-γ immunoreactivity was the marker most consistently identified in discs from patients with suspected discogenic pain compared to control subjects^70^.

Interferons recognized by their cognate receptors trigger signaling pathways leading to the activation of transcription of interferon-regulated genes. The outcome of the interferon response is determined by the nature and combination of these genes. All our patients had a disc herniation verified during the last two years before inclusion. Considering the aforementioned earlier findings of increased interferon expression after disc herniation, our observations may therefore be a result of (1) an increased interferon expression after disc herniation (that is not observable in our data as we did not have a control group without disc herniation) and (2) a subsequent deregulation of interferon-regulated genes, stimulating further inflammation, MC development and STIR signal increases. Nine of the in total 42 significantly differentially expressed genes found in our analyses were reported as interferon-regulated in the Interferome database. Seven of these showed the same direction in expression difference as expected after an interferon stimulation, further supporting this proposed mechanism.

The other gene sets that were found significantly enriched among the differentially expressed genes across all STIR variables were pathways related to defense response to virus and mitochondrial metabolism. The involvement of viruses in the degeneration and herniation of discs have been suggested, but studies show inconclusive results^71^. However, the genes underlying the defence to virus pathway have rather broad immune and inflammatory functions also occurring without a viral trigger.

The involvement of mitochondria, however, have more support. The dynamic structure and metabolism of mitochondria are known to affect the fate of cells, including regulating immune cell fate during immune responses^72^. Mitochondrial fission and fusion events where single mitochondria are separated into several structures or joined to larger ones, are of importance for T cell development toward memory or effector phenotypes^73^. Dysfunction in the dynamics of these events can also lead to the accumulation of damaged mitochondria, inducing oxidative stress and overproduction of reactive oxygen species (ROS). Such ROS are intracellular mediators that regulate antimicrobial and inflammatory responses. When dysregulated they can aggravate immune reactions and cause chronic inflammation^74^. Additionally, disc degeneration is thought to involve mitochondrial dysfunction, as fine-tuned cellular bioenergetics is fundamental to maintain matrix homeostasis in the intervertebral disc^75^.

Different cell types have different gene expression profiles, and thus the cell type proportions in blood samples could highly impact downstream gene expression analysis. As there were no correlations between the cell type proportions and the STIR variables, we did as expected not observe huge changes in the results when repeating the analyses adjusting for cell type proportions. Correspondingly, the GSEA results were also similar, but with fewer overlapping pathways between the three different STIR variable analyses. Adding more variables to the analysis model reduces the statistical power to detect significant associations, explaining the general reduction in significant findings.

Although the findings of this study were performed under fairly strict FDR control, we acknowledge the need for replication in other cohorts and in-depth studies to elucidate the biological mechanisms. In the present study, we chose to include only patients with MC type I, as this MC type is considered to mirror an active inflammatory state. The results would likely differ in a cohort with other MC types. This study was also restricted to patients with prior disc herniation, most of whom had marked DD. Our study did not include a control group without LBP, MC or disc herniation, as the participants were derived from a randomized controlled trial. A control group could have provided insight into whether genes were up-or down- regulated compared to a healthy state, and also whether our findings were driven by specific spine pathologies or were generalizable to the presence of STIR edema regardless of diagnosis.

In summary, we have demonstrated an association between STIR signals and systemic gene expression in patients with LBP and MC. Our results suggest that immunological processes, particularly interferon signaling, are important players in the underlying pathobiology of these patients.

## Supplementary Information

Supplementary Information 1.

Supplementary Information 2.

Supplementary Information 3.

Supplementary Information 4.

Supplementary Information 5.

Supplementary Information 6.

Supplementary Information 7.

Supplementary Information 8.

Supplementary Information 9.

## Data Availability

The datasets generated during and/or analyzed during the current study are available from the corresponding author on reasonable request in accordance with local registration and ethical approval.
