# Supplementary figures and images for: Correlation between gene expression and MRI STIR signals in patients with chronic low back pain and Modic changes indicates immune involvement
Source: Sci Rep. 2022 Jan 7;12:215. doi: 10.1038/s41598-021-04189-5 (PMC8741947; doi:10.1038/s41598-021-04189-5)

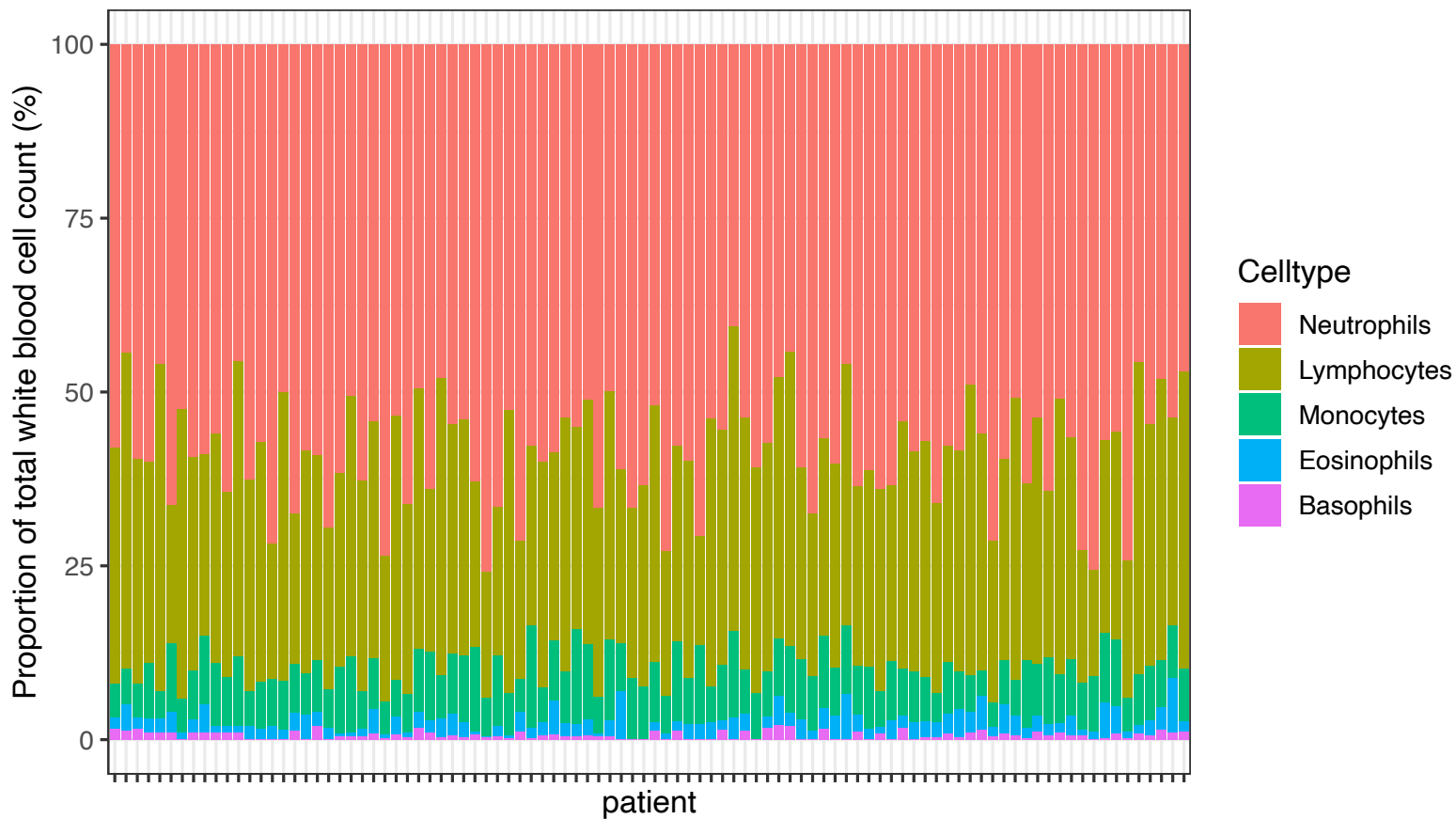

**Supplementary Figure 1: White blood cell type proportions (%) in 96 patients.**

Supplement: Supplementary file 1 — Supplementary Information 1. [file 41598_2021_4189_MOESM1_ESM.pdf]
