# Supplementary material for: Correlation between gene expression and MRI STIR signals in patients with chronic low back pain and Modic changes indicates immune involvement
Source: Sci Rep. 2022 Jan 7;12:215. doi: 10.1038/s41598-021-04189-5 (PMC8741947; doi:10.1038/s41598-021-04189-5)
Supplement: Supplementary file 2 — Supplementary Information 2. [file 41598_2021_4189_MOESM2_ESM.pdf]

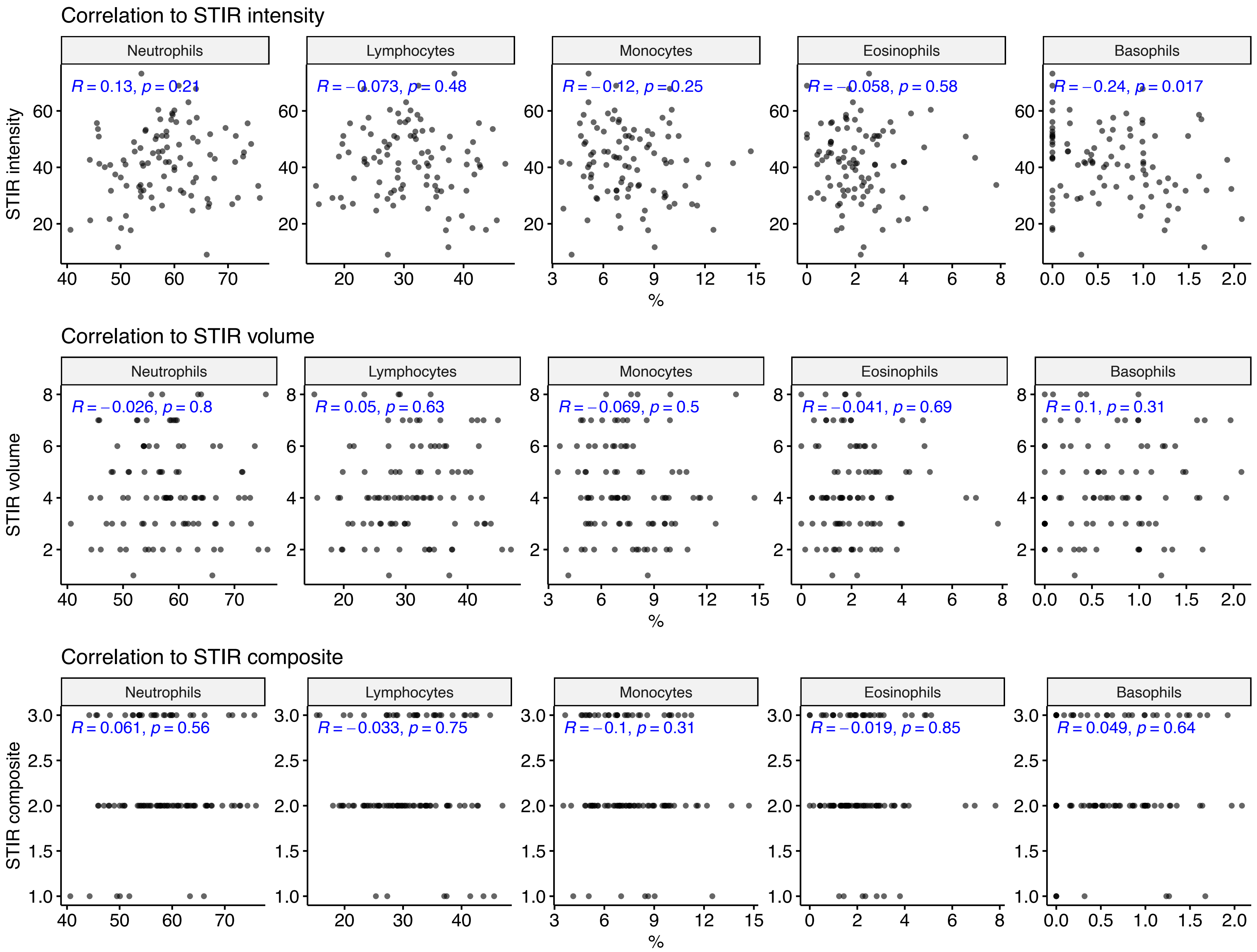

Supplementary Figure 2: Correlation of cell type proportions to A) STIR intensity, B) STIR volume, C) STIR composite
