# Supplementary material for: Correlation between gene expression and MRI STIR signals in patients with chronic low back pain and Modic changes indicates immune involvement
Source: Sci Rep. 2022 Jan 7;12:215. doi: 10.1038/s41598-021-04189-5 (PMC8741947; doi:10.1038/s41598-021-04189-5)
Supplement: Supplementary file 3 — Supplementary Information 3. [file 41598_2021_4189_MOESM3_ESM.pdf]

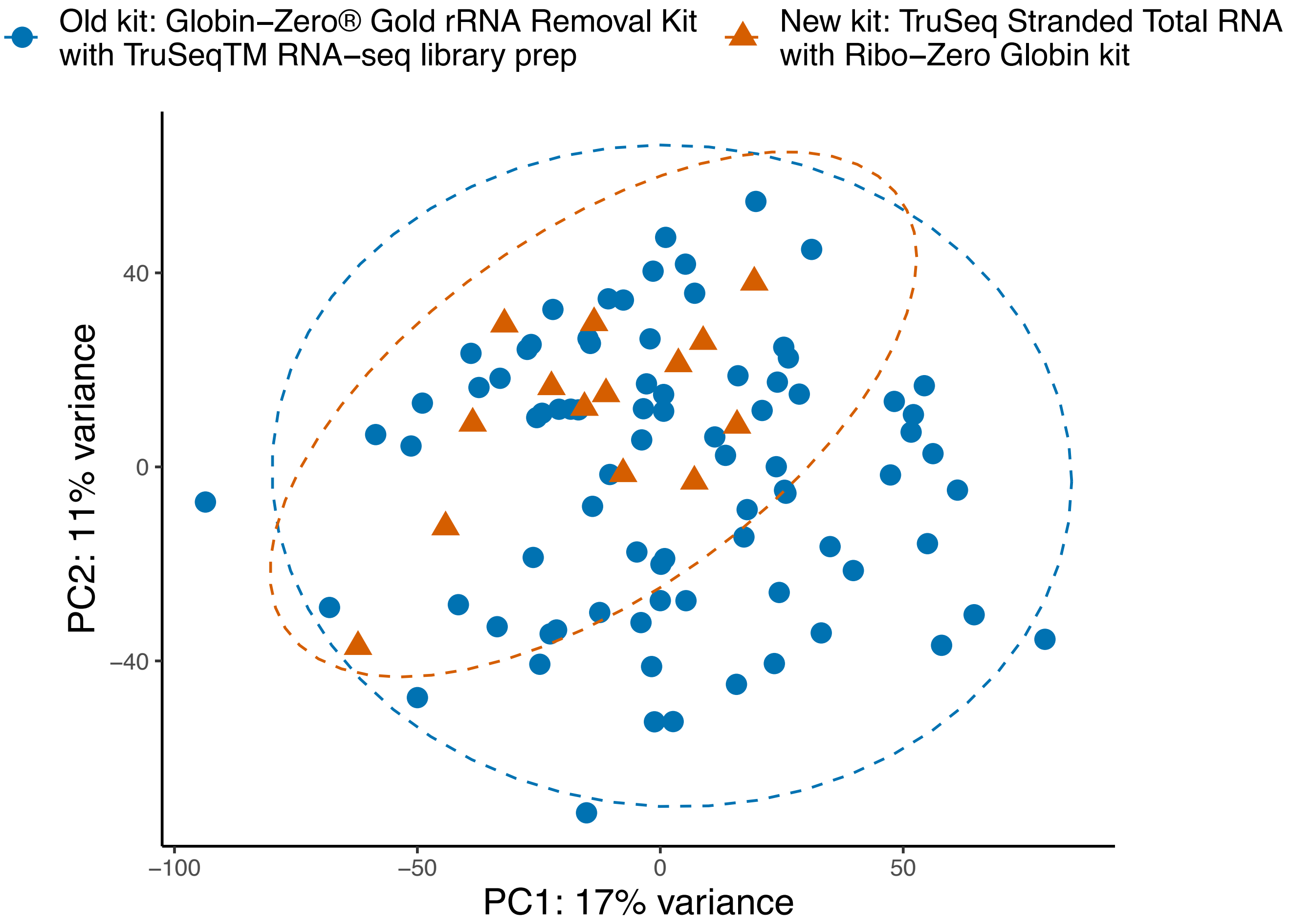

Supplementary Figure 3: Principal component analysis plot of the gene expression count data, demonstrating the variation between the samples. Each dot represents a sample, the colors/shapes represent the version of depletion kit used during library preparation. The counts are transformed using the varianceStabilizingTransformation function in DESeq2. PC = Principal component.
