# Supplementary material for: Correlation between gene expression and MRI STIR signals in patients with chronic low back pain and Modic changes indicates immune involvement
Source: Sci Rep. 2022 Jan 7;12:215. doi: 10.1038/s41598-021-04189-5 (PMC8741947; doi:10.1038/s41598-021-04189-5)
Supplement: Supplementary file 4 — Supplementary Information 4. [file 41598_2021_4189_MOESM4_ESM.pdf]

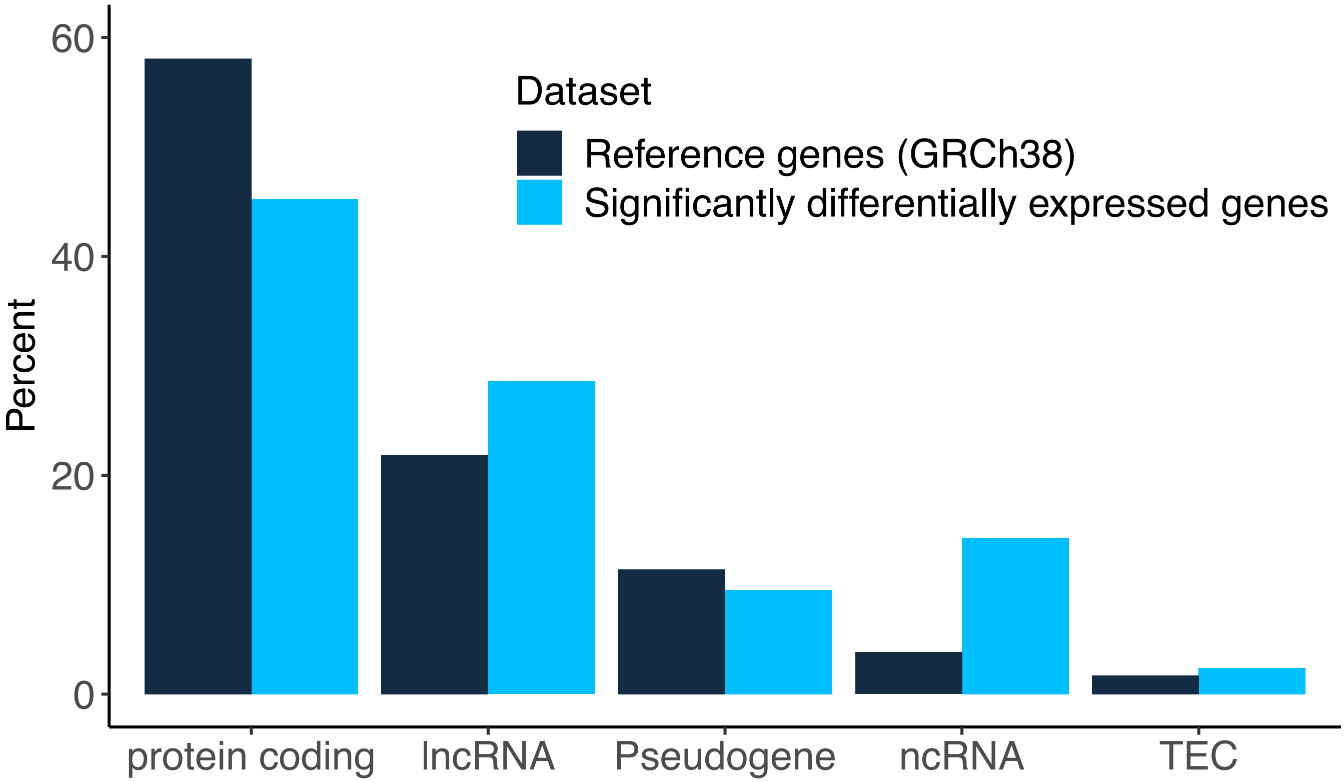

Supplementary Figure 4: Distribution of gene biotypes among the significantly differentially expressed genes (FDR < 0.05). ncRNA = non-coding RNA, lncRNA = long non-coding RNA, miRNA = microRNA.
