# Supplementary material for: Correlation between gene expression and MRI STIR signals in patients with chronic low back pain and Modic changes indicates immune involvement
Source: Sci Rep. 2022 Jan 7;12:215. doi: 10.1038/s41598-021-04189-5 (PMC8741947; doi:10.1038/s41598-021-04189-5)
Supplement: Supplementary file 5 — Supplementary Information 5. [file 41598_2021_4189_MOESM5_ESM.pdf]

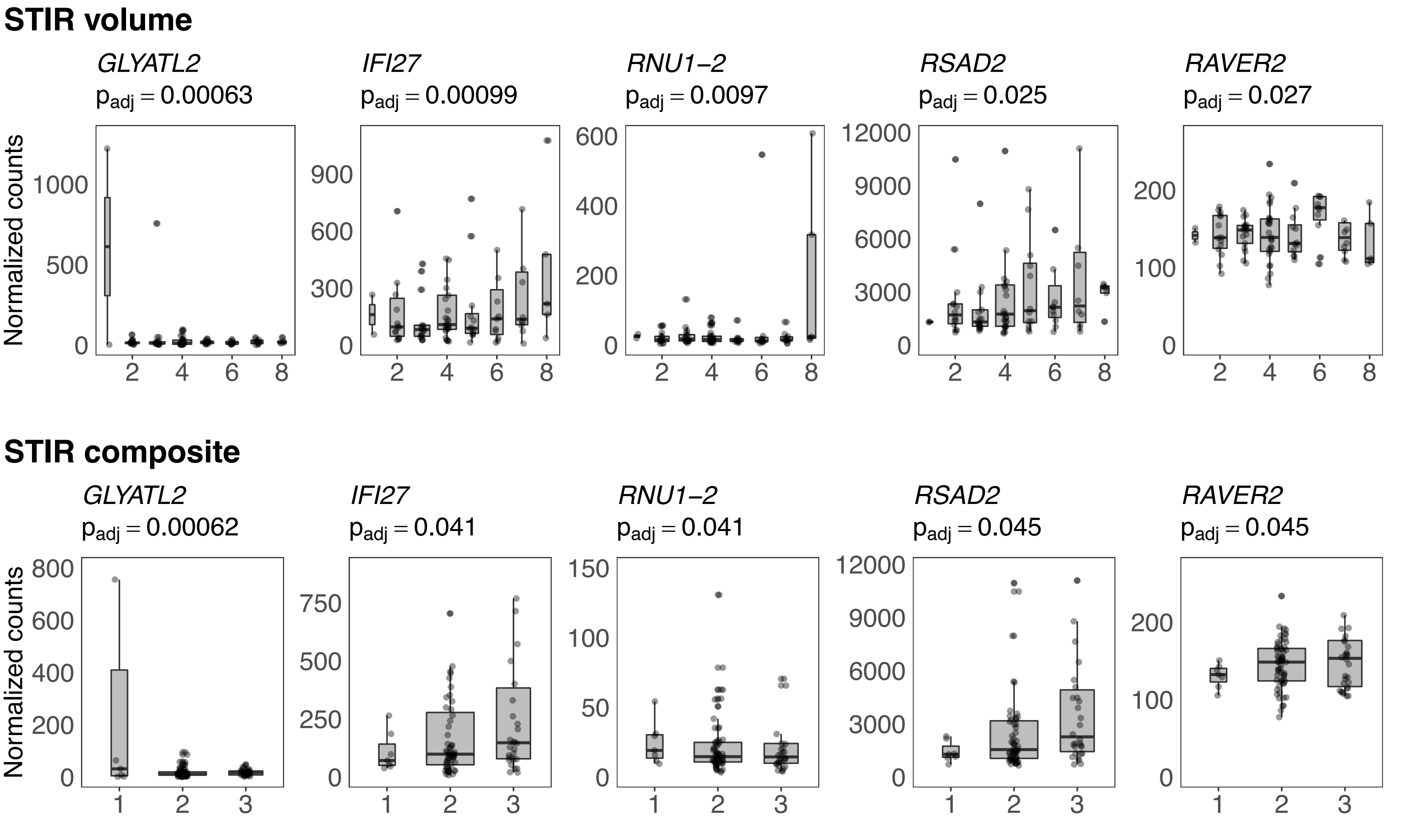

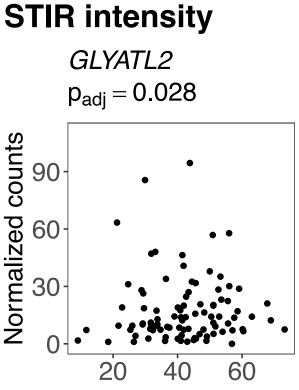

Supplementary Figure 5: Protein-coding genes significantly differentially expressed across analyses. Expression levels of the genes in each sample normalized by sequencing depth and RNA composition ("Normalized counts") vs STIR variable values. P-values are the adjusted significance values from the differential expression analyses (Table S2-S4).
