# Supplementary material for: Correlation between gene expression and MRI STIR signals in patients with chronic low back pain and Modic changes indicates immune involvement
Source: Sci Rep. 2022 Jan 7;12:215. doi: 10.1038/s41598-021-04189-5 (PMC8741947; doi:10.1038/s41598-021-04189-5)
Supplement: Supplementary file 6 — Supplementary Information 6. [file 41598_2021_4189_MOESM6_ESM.pdf]

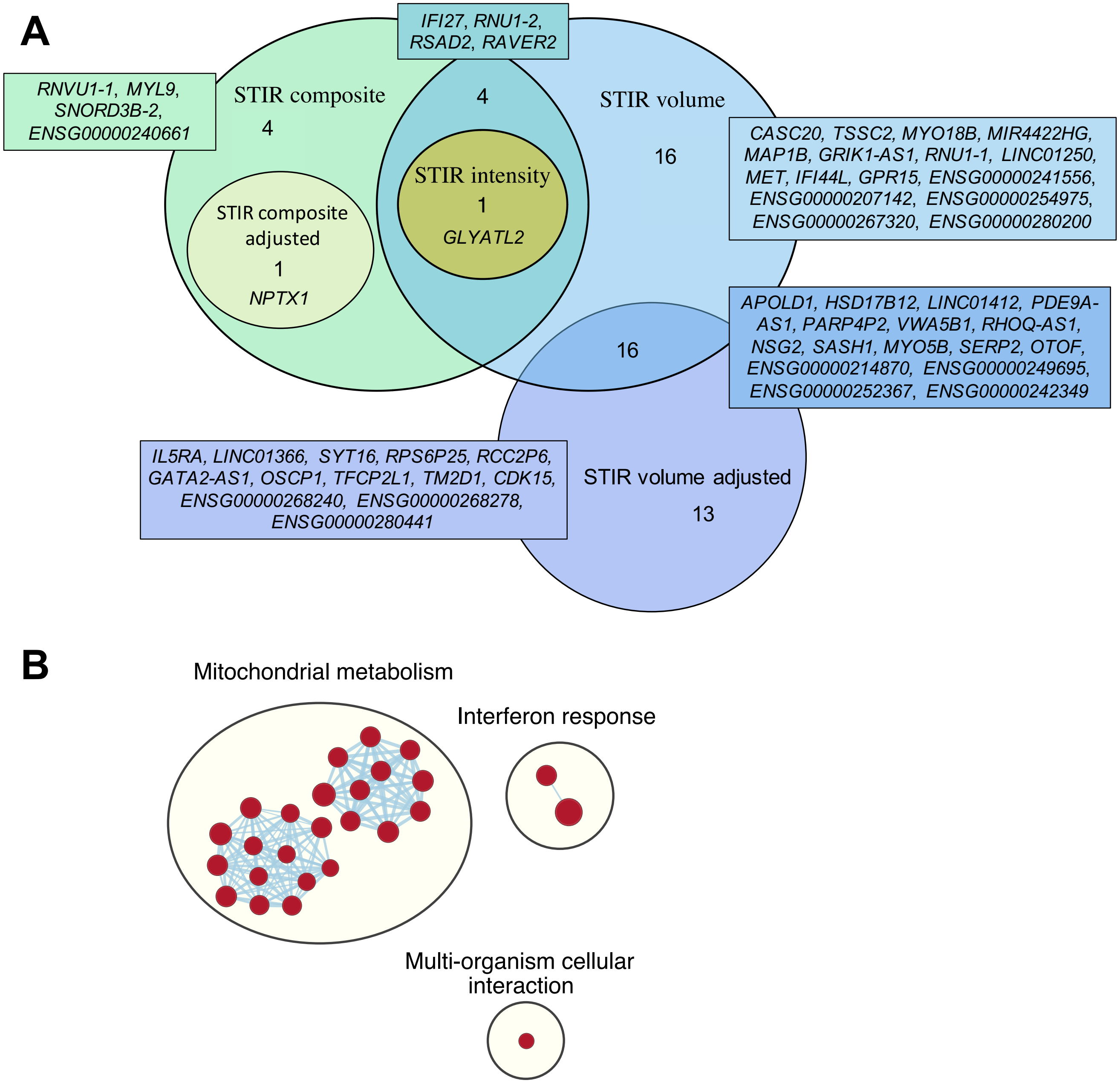

Supplementary Figure 6: Results from analyses adjusted for cell type proportions (FDR < 5%). A) Venn diagram of overlapping DE genes from all analyses, including analyses with and without adjustment for cell type proportions ("adjusted"). STIR intensity adjusted show no significant DE genes, and is therefore not represented in the illustration. B) Gene sets significantly enriched among differentially expressed genes across all adjusted analyses, visualized using Cytoscape. Pathways related to mitochondrial metabolism, interferon response, and multi-organism cellular interaction were significantly enriched among the upregulated genes in all three analyses. Each pathway is represented by a red node, overlaps of genes between the pathways are shown as light blue edges. The node size represents the number of genes in the gene set.
